# What do adolescents want in order to become more active?

**DOI:** 10.1186/1471-2458-13-718

**Published:** 2013-08-05

**Authors:** Kirsten Corder, Andrew J Atkin, Ulf Ekelund, Esther MF van Sluijs

**Affiliations:** 1 MRC Epidemiology Unit, University of Cambridge Addenbrookes Hospital, Box 285, Hills Road, Cambridge CB2 0QQ, UK; 2 UKCRC Centre for Diet and Activity Research (CEDAR), Institute of Public Health, University of Cambridge, Box 296, Forvie Site, Robinson Way, Cambridge CB2 0SR, UK; 3 Department of Sports Medicine, Norwegian School of Sports Sciences, PO Box 4014, Ullevål Stadion, 0806, Oslo, Norway

## Abstract

**Background:**

Few large studies have examined adolescents’ views about increasing their physical activity (PA) to inform PA promotion. We assessed adolescent preference for activity type, co-participants, timing and location of PA promotion and examined patterns in their views by population subgroup.

**Methods:**

Participants (n = 457) (Mean ± SD age: 14.3 ± 0.3 years; 45.2% male) responded to questionnaire items: “What activities would you like to try or do more often?” (yes/no to 6 activity types e.g. team sports) and “I would like to do more PA …” followed by options regarding co-participants, timing and PA location (agree/disagree to 10 items). Anthropometry, demographics, accelerometer- and questionnaire-derived PA were obtained. Logistic regression was used to examine differences in views by subgroup (sex, weight status, objective PA level, parental education (SES)).

**Results:**

Most adolescents wanted to increase participation in ≥1 type of PA (94.4%). Gym use (56.7%) and team sports (50.6%) were most popular. Girls were less likely to choose racquet sports (vs. boys OR; 95% CI 0.6;0.4-0.9) but more likely to select dancing (40.3;17.8-91.1). Preference for participation was positively associated with existing participation in a similar activity (all p < 0.02). More adolescents wanted to increase PA with friends (88.8%) than family (63.5%). A leisure centre was most popular for increased participation (81.0%), followed by home (70.0%). Participation during school time was less popular among girls (vs. boys: 0.6;0.4-0.9) and more popular among low SES participants (vs. high: 1.6;1.1-2.4). Overweight/obese adolescents were less likely to choose participation with friends (vs. normal weight 0.5;0.3-0.9).

**Conclusions:**

Targeting adolescent PA promotion by subgroup and providing choice of PA type, co-participants, timing and PA location appears promising. Adolescents want to do more types of PA more often; interventions could increase opportunities and support to facilitate this.

## Background

Insufficient physical activity is a risk factor for obesity and related metabolic disorders in youth [1] and physical activity declines throughout adolescence [2]. Adolescence is, therefore, an important period for physical activity promotion and aiming to attenuate the age-related decline in physical activity [3-6]. There is extensive cross-sectional evidence examining factors associated with physical activity among adolescents [7-9] and an increasing amount using longitudinal data [10]. In a review of factors prospectively associated with change in physical activity, few consistent factors were identified with which to inform intervention development [10]. It is, therefore, perhaps unsurprising that reviews highlight the limited success of physical activity promotion in youth [11-14].

Relatively little research has utilised adolescents’ views to develop and inform physical activity promotion [15]. Autonomy for many behaviours increases during adolescence and therefore involving adolescents in intervention development appears to be developmentally appropriate [16]. Qualitative research has considered adolescent opinion in physical activity promotion research but typically only in small samples. To our knowledge, most previous qualitative research has been specific to existing physical activity promotion programmes [17] , has investigated one population group e.g. girls [17,18] or has investigated only specific factors such as gender preferences [19] with the results therefore specific to those situations and not adolescents in general.

Offering a variety of activities may be important for adolescent physical activity promotion [18,19] but, to our knowledge, little is known about what types of activities would be appealing to specific population subgroups. Epidemiological data shows differential participation in physical activities according to gender stereotypes, for example higher participation of girls in aerobics and dancing, and markedly less female participation in basketball and football [20]. It is possible that these differences could be due to opportunity rather than choice. Currently, it is unknown which activities different subgroups of adolescents would like to participate in more frequently. This information could help to improve targeting of physical activity promotion strategies.

Additionally, little is known about adolescent opinion regarding other aspects of physical activity promotion. It is often suggested that parental influence on physical activity decreases during adolescence, as peer influence becomes more important, but it is uncertain to what extent the importance of peer support surpasses family support for physical activity as children age [21]. Furthermore, adolescents use a large variety of places for their physical activity [22] but there is very little evidence as to which places are appealing for adolescents to do more physical activity. During adolescence, physical activity declines most during weekends and after school [23] but interventions are often targeted during school time, perhaps for convenience of researchers in terms of recruitment [24]. However, it is unclear whether this is most suitable for adolescents themselves. To our knowledge, adolescent preference for co-participants, timing and location of additional physical activity opportunities has not been investigated in a large study and could be helpful for informing intervention development.

We aimed to investigate what activities adolescents would like to do more often, and when, where and with whom adolescents would like to be more active. This was an exploratory study; however, we hypothesised that adolescent preference for physical activity type and location may differ across social and demographic subgroups (defined by sex, weight status, objective physical activity level, parental education (SES)).

## Methods

### Study design and setting

The SPEEDY study (Sport, Physical activity and Eating behaviour: Environmental Determinants in Young people) is a population-based longitudinal cohort study, investigating factors associated with physical activity and dietary behaviour among children attending schools in the county of Norfolk, UK [25]. Full details on participant recruitment and study procedures for baseline data collection have been described elsewhere [25]. Ethical approval for the whole study was obtained from the University of East Anglia research ethics committee.

### Participant recruitment

Participants were invited to be measured on three separate occasions: baseline (age 9/10y; April-July 2007), +1 year (age 10/11y; April–July 2008) and +4 years (age 13/14y; April-August 2011). Briefly, at baseline, schools in Norfolk were purposively sampled to achieve urban and rural heterogeneity. From 227 eligible schools (those with ≥12 Year 5 children (9/10y)), 157 were approached and 92 schools were recruited. All Year 5 children (n = 3619) at the 92 schools were invited to participate. In total, 2064 children provided parental consent to participate and were measured at baseline (57% response rate). As reported previously [26], of 2064 baseline participants invited to 1-year follow-up, 1019 (49.4% of original sample) consented and of these, 954 (46.2%) provided data. All participants with valid home addresses after 1-year follow-up (n = 1964) were invited to take part in +4 year-follow-up. Our original consent did not allow us to trace individual participants through their schools so we presented the study at Year 9 assemblies (13/14y) at Norfolk secondary schools to encourage previous participants to take part. Due to low recruitment (21.4% of invited participants), an extra invitation letter was sent home at the end of the school term (July 2011) inviting participants to be assessed by mail over the school summer holidays.

### Data collection procedures

Participants consenting to four year follow-up during the school term were visited at school by researchers where anthropometric measures were taken, participants completed questionnaires and were fitted with accelerometers which were returned to school one week later. Parents were mailed a questionnaire which they were asked to complete and return. Participants providing parental consent for assessment during the summer holidays (n = 62) were mailed adolescent and parent questionnaires, and an accelerometer and instruction sheet requesting that they wear the monitor for one week, complete the questionnaires and return them by mail. Parents of students assessed during the summer holidays were additionally asked to report their son/daughter’s height and weight in the parent questionnaire.

Data in this paper are from the third measurement of this cohort in 2011 when participants were (mean (SD)) 14.3 (0.3) years-old unless otherwise stated.

### Outcome variables

Outcome variables were derived from questions included in the adolescent questionnaire. These variables did not refer to a specific timeframe and included the example activities as stated below. Participants were asked to “tick all that apply” for “Which of these activities or sports would you like to try or do more often?” with a list of six broad activity groups (fitness classes (e.g. aerobics, pilates yoga), dancing (e.g. hip hop, ballet, ballroom), martial arts (e.g. judo, karate, aikido), racquet sports (e.g. badminton, squash), team sports (e.g. rugby, netball), using a gym (e.g. treadmills, weights)). An ‘other’ category with a free text field and an option for “I don’t want to do any more activities or sports” were also included. This selection was based on discussion with scientific peers and piloted among adolescents within the target age group. Responses for each broad activity group were summarised as yes or no for each participant (coded 1 and 0 respectively). Activities included in the ‘other’ category were recoded for inclusion in the broad activity groups where relevant. Due to the remaining relatively small number of ‘other activities’ (n = 48) including many unique entries, these were not included in regression analyses as outcome variables. A continuous variable regarding the number of activities adolescents would like to do more often was derived as a sum of all activity types selected; the responses were coded as yes or no for each activity type and the continuous variable was a sum of the yes responses.

To assess adolescent preference for when, where and with whom they would like to be more active, participants were asked whether they agreed with the following statement: “I would like to do more physical activities or sports …”. Individual response items regarded with whom (family, friends), when (during school time, weekends, after school), and where (home/garden, community centre, local park, school grounds, gym/leisure centre). Response categories for each item were strongly agree, agree, disagree and strongly disagree, recoded as agree (strongly agree and agree) or disagree (disagree and strongly disagree), coded 1 and 0 respectively. These items referred to any activity or sport and not just the six activity types investigated here.

### Objective physical activity measurement

Physical activity was objectively assessed using the Actigraph accelerometer (Model GT1M). The Actigraph has been shown to accurately assess energy expenditure among European children during free-living conditions [27,28]. The monitor was set to record at 5-second epochs. Participants were asked to wear the monitors during waking hours for 7 days and to remove them whilst bathing, showering and swimming.

Accelerometry data were analyzed using a batch processing program (MAHUffe: http://www.mrc-epid.cam.ac.uk/Research/PA/Downloads.html) to remove any data recorded after 11 pm and before 6 am. Periods of ten minutes or more that had continuous zero activity counts [4,29,30] and any days with less than 500 minutes of recording [30] were excluded. All other days were included in analyses.

Data were examined as time (min/d) spent in MVPA, derived using 2000 (Actigraph) counts/min as the lower threshold [31,32]. Participants were then classified as ‘meeting physical activity recommendations’ or not using a threshold of an average of 60 min/d MVPA, according to UK physical activity recommendations [33,34].

### Demographic and biological exposures

Adolescent age and gender were assessed during the measurement session. Standardized protocols were used to measure height and weight. Height was measured to the nearest millimetre (Leicester height measure, Chasmors Ltd., Leicester, UK). A non-segmental bio-impedance scale was used to measure weight (to the nearest 0.1 kilogram) and impedance in light clothing (Tanita, type TBF-300A. Tokyo, Japan). Height and weight was parentally reported for 57 of the 62 participants who were assessed by mail during the school summer holidays. Both measured and parent-reported height and weight were used to calculate body mass index (BMI, kg/m^2^). BMI z-scores and subsequently overweight and obesity status were derived using sex- and age-dependent cut points [35].

Variables parentally reported at baseline were used to determine ethnicity and parental education. Paternal and maternal ethnicity were reported separately for both parents and used to establish child’s ethnicity; 96.1% of the sample was white so this variable was not included in the analysis. The main caregiver self-reported their age at leaving full time education in three categories which were dichotomized into ≤16 years, and >16 years of age and coded 0 and 1 respectively.

To aid interpretation of results, all subgroup variables (sex, weight status, physical activity level, parent education) were recoded with 1 as the group hypothesized to be in most need of physical activity promotion: girls, overweight/obese, not meeting physical activity recommendations and low parental education.

### Self-reported physical activity

The validated Youth Physical Activity Questionnaire (YPAQ) was administered at the measurement session [36] and was based on the Children’s Leisure Activities Study Survey (CLASS) [37]. The YPAQ lists 47 different activities with participants requested to self-report the frequency and duration of each activity over the last 7 days. Participants could select participation in as many activities as they liked. This data was used to establish current participation in activities aligned to the broad activity group outcome variables except for gym use which was not included on the self-report questionnaire (coded 0 as not participating and 1 as participating). Participants assessed during the school summer holidays (n = 62) did not complete this questionnaire.

### Statistics

Differences in the baseline characteristics of participants with (n = 457) and without (n = 1612) valid outcome data at 4-year follow-up were tested using t-tests or chi-squared tests for continuous and categorical variables, respectively.

Differences in participant characteristics by sex were tested using t-tests or chi chi-squared tests for continuous and categorical variables, respectively.

Outcome variables were dichotomous items regarding the activities adolescents would like to try or do more often, and with whom, when and where they would like to do more physical activities or sports. A continuous variable regarding the number of activities adolescents would like to do more often was also included as an outcome variable.

Multilevel logistic regression was used to test differences in each outcome variable by subgroup (sex, weight status, physical activity level, parent education). Multilevel linear regression was used for the continuous outcome variable. Multilevel logistic regression was also used to determine whether wanting to try or do an activity type more often was associated with current participation in that activity type as derived from the self-report questionnaire. All regression analyses were clustered for school attended at follow-up.

Sensitivity analyses were carried out excluding participants assessed during the summer holidays and for the 18% of participants not having any weekend days of physical activity data. Result did not vary; therefore results from the whole sample with data at 4 year follow-up measurements are included.

Analyses were carried out using Stata 12.0 (Statacorp, College Station, TX).

## Results

All 1964 (95.2%) baseline participants with valid contact details after 1-year follow-up were invited to take part in 4-year follow-up. Of these, 480 consented and 457 (22.1%) provided data for all outcome and demographic variables. Self-report PA data was provided by 402 participants, 410 provided valid accelerometer data for at least one day and 377 participants had valid data for all variables. The analytical sample (n = 457) did not differ by baseline age (p = 0.46), BMI z-score (p = 0.51), MVPA (p = 0.71) or sex (p = 0.88) compared to those in the baseline study who did not provide outcome data for this analysis.

Participant characteristics for the 457 participants included in these analyses are presented in Table 1. The accelerometer data suggests that boys were more active than girls based on both minutes of MVPA (p < 0.001) and percentage meeting the physical activity guideline (p = 0.013). Parents of boys had higher education than parents of girls (p = 0.04). No other sex differences were identified.

**Table 1:** Descriptive characteristics of 457 participants included in the final analyses

**Demographic characteristics**	**All N = 457**	**Boys N = 218**	**Girls N = 262**	**p**
**Age** (years) Mean (SD)	14.3 (0.3)	14.3 (0.3)	14.3 (0.3)	0.11
**BMI Z-score** (kg · m^-2^) Mean (SD)	0.37 (1.2)	0.29 (1.19)	0.43 (1.22)	0.25
**Weight status** N (% overweight / obese)	96 (21.0)	38 (18.3)	58 (23.3)	0.19
**Parent education** N (%)				0.04
*Low*	179 (39.2)	71 (34.1)	108 (43.4)	
*High*	278 (60.8)	137 (65.9)	141 (56.6)	
**Objective physical activity**	**All** N = 410	**Boys** N = 192	**Girls** N = 218	**p**
**MVPA** (min · d^-1^) Mean (SD)	62.8 (24.7)	67.3 (25.6)	58.8 (23.2)	<0.001
**Meeting PA Guidelines** N (% meeting guideline)	248 (54.3)	126 (60.6)	122 (49.0)	0.013

Students T-test of chi-squared tests used to assess sex differences.

MVPA moderate and vigorous physical activity.

Meeting PA Guidelines defined as ≥ 60 minutes per day of MVPA (≥2000 accelerometry counts per minute).

Descriptive data for all outcome variables are presented in Table 2. Overall, gym use (e.g. treadmills/weights) (56.5%) and team sports (50.1%) were most popular for increased participation. Most adolescents wanted to increase participation in ≥1 type of physical activity (89.7%). The mean (SD) number of activities that adolescents wanted to try, or do more often was 2.5 (1.3).

**Table 2:** Descriptive data for outcome variables (N = 457) regarding what activities adolescents would like to try or do more often and with who, when and where adolescents would like to do more physical activity

**Which of these activities or sports would you**	**N Yes**	**% Yes**
**like to try or do more often?**		
Team sports (e.g. rugby, netball)	229	50.1
Fitness classes (e.g. aerobics, pilates, yoga)	128	28.0
Racquet sports (e.g. badminton, squash)	192	42.0
Dance (e.g. hip hop, ballet, ballroom)	139	30.4
Martial arts (e.g. judo, karate, aikido)	94	20.6
Using a gym (e.g. treadmills, weights)	258	56.5
Other	78	17.1
None (I don’t want to do any more physical activities or sports)	47	10.3
**Number of activities**	**N**	**%**
0	47	10.3
1	63	13.8
2	135	29.5
3	114	25.0
4	75	16.4
5	17	3.7
6	4	0.9
7	2	0.4
≥1	410	89.7
	**Mean**	**SD**
Total number of activities	2.5	1.3
**I would like to do more physical activities**	**N agree**	**% agree**
**or sports…**		
**With (Co-participation)**		
With my friends	406	88.8
With my family	290	63.5
**When (Timing)**		
During school	337	73.7
At weekends	346	75.7
After school	345	75.5
**Where (Location)**		
At home/in the garden	320	70.0
At a local community centre	194	42.5
At a local park	290	63.5
In school grounds	291	63.7
At a gym/leisure centre	370	81.0

N Yes: number of participants answering yes to that question.

Number of activities: Number of separate yes responses to types of activities participants wanted to try or participate in more often.

Total number of activities: Mean (SD) of number of activities.

N agree: number of participants answering agree or strongly agree.

%: Percentage of sample responding yes or agreeing to questionnaire responses.

Descriptive results show that 88.8% of adolescents want to do more physical activity with friends and 63.5% with their family. The majority of adolescents wanted to do more physical activity or sports during school time (73.7%), at weekends (75.7%) and after school (75.5%). A gym/leisure centre was the most popular choice for increased participation (81.0%), followed by at home/in the garden (70.0%). A local community centre was the least favoured choice (42.5%) for increased participation.

Results from multilevel logistic regression examining differences in which activities or sports adolescents would like to try or do more often by population subgroup are presented in Table 3. Preferences regarding type of activity for increased participation differed by sex, activity level and parental education, but not weight status. To increase physical activity, girls were less likely to choose racquet sports (vs. boys) (OR (95% CI) p value: 0.6 (0.4, 0.9) <0.001) but more likely to select dancing (40.3 (17.8, 91.1) p < 0.001) or fitness classes (7.2 (3.9, 13.3) p < 0.001). Participants not meeting physical activity guidelines were less likely to want to use a gym (0.6 (0.4, 0.9) p = 0.03). Participants with low parental education were less likely to choose racquet sports (0.5 (0.5, 0.9) p = 0.013) and more likely to choose dance (1.7 (1.2, 2.5) p = 0.003). Preference to participate in a broad activity group was positively associated with already participating in an activity within that group over the previous week (all p < 0.02 where available). Being a girl (0.4 (0.2, 0.9) p < 0.001), having parents with low education (1.9 (1.0, 3.6) p = 0.04) and not reporting doing any activities (9.9 (1.6, 60.6) p = 0.01) were associated with not wanting to try or do any more activities more often.

**Table 3:** Results from multi-level regression clustered for school at follow-up examining differences in adolescents’ views about type of activity for increased participation by broad population subgroup

**Which of these activities**	**Girls (vs. boys)**	**Overweight/Obese**	**<60 mins/day MVPA**	**Low parent**	**N (%) already**	**Already**
**or sports would you**		**(vs. normal weight)**	**(vs. ≥60 mins/day**	**education**	**participate**	**participate**
**like to try or do**			**MVPA)**	**(vs. high)**	**(N = 402)**	**(Yes vs. No)**
**more often?**						**(N = 402)**
	**Multiple logistic regression OR (95% CI)**					
**Team sports** (e.g. rugby, netball)	0.7 (0.5, 1.2)	0.7 (0.5, 1.2)	0.9 (0.6, 1.4)	0.7 (0.5, 1.1)	246 (53.8)	**3.9 (2.6, 5.9)**
**Fitness classes** (e.g. aerobics, pilates, yoga)	**7.2 (3.9, 13.3)**	1.2 (0.8, 1.8)	1.1 (0.6, 1.9)	0.7 (0.4, 1.1)	21 (4.6)	**3.5 (1.5, 8.1)**
**Racquet sports** (e.g. badminton, squash)	**0.6 (0.4, 0.9)**	0.8 (0.5, 1.4)	1.0 (0.6, 1.5)	**0.47 (0.5, 0.9)**	104 (22.8)	**4.1 (2.5, 6.6)**
**Dance** (e.g. hip hop, ballet, ballroom)	**40.3 (17.8, 91.1)**	1.5 (0.9, 2.3)	1.2 (0.7, 1.8)	**1.7 (1.2, 2.5)**	84 (18.4)	**8.1 (5.4, 12.1)**
**Martial arts** (e.g. judo, karate, aikido)	0.7 (0.4, 1.1)	0.9 (0.5, 1.6)	0.6 (0.4, 1.1)	0.9 (0.5, 1.4)	28 (6.1)	**5.0 (2.1, 11.7)**
**Using a gym** (e.g. treadmills, weights)	1.0 (0.7, 1.6)	1.1 (0.6, 2.0)	**0.6 (0.4, 0.9)**	1.0 (0.7, 1.5)	0 (0)	
**None** (I don’t want to do any more physical activities or sports)	**0.4 (0.2, 0.9)**	0.5 (0.2, 1.7)	1.9 (0.9, 3.9)	**1.9 (1.0, 3.6)**	6 (1.3)	**9.9 (1.6, 60.6)**
	**Multiple linear regression B (95% CI)**					
**Total number of activities**	**0.6 (0.3, 0.9)**	0.04 (−0.3, 0.4)	−0.2 (−0.5, 0.09)	−0.15 (−0.39, 0.09)	2.7 (1.6)	**0.37 (0.01, 0.73)**

MVPA: moderate and vigorous physical activity defined ≥2000 accelerometry counts per minute.

Already participate: Number and percentage of participants who report already participating in an activity within that group.

Total number of activities: Mean (SD) of number of activity types which adolescents would like try or do more often.

Results from multilevel logistic regression examining subgroup differences for adolescent views on co-participants, timing and location of increased physical activity are presented in Table 4. Differences by subgroup were seen for co-participants, timing and location of physical activity. Girls (vs. boys) were less likely to want to participate during school time (OR 95% CI p value: 0.6 (0.4, 0.9) p = 0.03) whereas overweight/obese adolescents (vs. normal weight) were less likely to choose participation with friends (0.4 (0.2, 0.6) p < 0.001). Those not meeting physical activity recommendations were less likely to want to participate in more activities at all locations other than at home (all p < 0.04). Participants with low parental education were more likely to want to participate during school time (1.6 (1.1, 2.4) p = 0.03) and less likely to choose a gym/leisure centre (0.6 (0.3, 0.9) p = 0.025).

**Table 4:** Results from multi-level logistic regression clustered for school at follow-up examining differences in adolescents’ views about co-participants, timing and location of increased activity participation by broad population subgroup

**I would like to do more**	**Girls (vs. boys)**	**Overweight/Obese**	**<60 mins/day MVPA**	**Low parent education**
**physical activities or sports…**		**(vs. normal weight)**	**(vs. ≥60 mins/day MVPA)**	**(vs. high)**
**With (Co-participation)**				
With my family	1.5 (0.9, 2.6)	1.1 (0.7, 1.9)	1.0 (0.7, 1.4)	1.1 (0.6, 1.8)
With my friends	1.0 (0.5, 1.9)	**0.4 (0.2, 0.6)**	0.6 (0.3, 1.1)	1.6 (0.8, 3.0)
**When (Timing)**				
During school time	**0.6 (0.4, 0.9)**	1.0 (0.6, 1.7)	0.7 (0.5, 1.2)	**1.6 (1.1, 2.4)**
At weekends	0.9 (0.6, 1.3)	1.4 (0.8, 2.5)	0.9 (0.6, 1.3)	0.8 (0.5, 1.4)
After school	0.8 (0.5, 1.4)	0.7 (0.4, 1.2)	0.7 (0.4, 1.1)	1.0 (0.6, 1.7)
**Where (Location)**				
At home/ in the garden	1.3 (0.8, 1.9)	1.1 (0.7, 1.9)	0.9 (0.6, 1.2)	0.9 (0.6, 1.5)
At a local community centre	1.1 (0.7, 1.6)	1.0 (0.6, 1.7)	**0.5 (0.3, 0.7)**	1.0 (0.7, 1.4)
At a local park	**0.5 (0.3, 0.9)**	**0.6 (0.4, 0.9)**	**0.6 (0.4, 0.9)**	1.1 (0.8, 1.6)
In school grounds	0.7 (0.5, 1.1)	0.8 (0.5, 1.4)	**0.5 (0.3, 0.8)**	1.2 (0.9, 1.6)
At a gym/leisure centre	0.8 (0.5, 1.3)	1.0 (0.5, 1.8)	**0.3 (0.2, 0.5)**	**0.6 (0.3, 0.9)**

Results are OR (95% CI).

MVPA: moderate and vigorous physical activity defined ≥2000 accelerometry counts per minute.

## Discussion

Adolescents appear to want to do more types of physical activities more often and interventions could increase opportunities and support to facilitate this. Specific targeting by population subgroup, incorporating choice of activity type, co-participants, timing and location of physical activity, may have potential for adolescent physical activity promotion. Researchers could focus on developing innovative ways to introduce adolescents to new activities and to facilitate opportunities for them to do more of activities that they currently participate in. Introducing adolescents to new activities may be especially important as once adolescents have tried an activity type they may be more likely to want to do more of it.

Adolescent views regarding increased physical activity participation differed by population subgroups, which highlights the potential of targeting interventions for specific groups. This is also aligned with calls in the literature for studies specifically targeting at risk groups, such as those with low physical activity levels [38]. However, evidence for the effectiveness of adolescent physical activity promotion interventions stratified by subgroup is currently inconclusive [12]. Nevertheless, our results provide some general pointers for targeting promotion by the subgroups explored in this study. Low socio-economic participants were more likely to want to participate during school time and were less likely to have a preference for increased participation in gyms or leisure centres. Cost has been shown as a barrier to physical activity among adolescents [39] and activities at school may be lower cost than at gyms and leisure centres. Therefore, creating no-cost opportunities for physical activity is vital.

Although stratification or tailoring an intervention for a specific population subgroup could provide a starting point for a physical activity intervention, it is unlikely to be sufficient as activity preferences also vary on an individual level. Although girls were much more likely than boys to favour increased participation in dancing, many girls also wanted to do more team sports, and other evidence suggests that girls can become frustrated with the gender stereotypes in physical education classes [40]. These adolescents reported wanting to do an additional 2.5 (1.3) types of activity more often, which also supports the incorporation of individual choice and variety into intervention design, as highlighted in previous qualitative research [15,18,19,40-42]. Despite this evidence advocating the need to incorporate choice in adolescent physical activity promotion programs, relatively little intervention work has done this [15]. This could, at least partly, be due to logistical reasons, as providing choice will inevitably make promotion programmes inherently more complicated to design, implement and evaluate.

Allowing for individual choice regarding co-participation, timing and location of physical activity may also be particularly relevant to specific population groups. Overweight and obese adolescents were less likely to want to participate in more activities with friends. Having to show others an unfit body, and lacking confidence and competence in core skills have previously been identified as barriers to physical activity among adolescent girls [42]. Therefore, offering alternatives for physical activity co-participation which do not necessarily involve peer groups may be valuable. This strategy could include promoting activities that could be done individually or perhaps with families. Despite popular belief about adolescents’ relationships with their families, 60% of these adolescents still wanted to do more physical activity with their family. Providing adolescents with choice and flexibility regarding these contextual intervention aspects would also allow sensitivity to adolescents’ individual development surrounding social relationships and physical maturity [41]. Future studies may wish to investigate these views in reference to other groups of people not investigated here, such as classmates or specific family members.

Adolescents were more likely to want to try an activity or do it more often if they reported already doing a similar activity. One could hypothesise that this could be due to perceived competence, confidence or self-efficacy. Qualitative evidence suggests that adolescents feel uncomfortable in physical education classes if they do not have the skills to be proficient and that enjoyment is linked to perceived competence [41]. Our results suggest that this issue may extend from activity type to other physical activity contexts as adolescents not meeting physical activity recommendations were less likely to choose to increase participation in locations outside of home. Although we are unable to ascertain whether these participants already do activity at these places, it is possible that familiarity of an environment for physical activity may play a role. Perhaps finding appealing ways to introduce adolescents to new activities and new physical activity locations could be important for more successful adolescent physical activity promotion. Introducing adolescents to new activities and locations may be especially important as once adolescents have tried an activity type, or been active in a particular location, they may be more likely to want to repeat this.

Our results indicate that most of these adolescents (94.1%) want to do more types of activity. Despite this apparent willingness to increase physical activity, most physical activity promotion strategies are not effective [12,24,43,44]. Although an adolescent may say that they would like to try, or do more of a certain activity, it may not mean that they would take an opportunity if it was presented to them. Therefore, more work is necessary to translate this apparent enthusiasm for increased physical activity participation into effective physical activity promotion interventions. A possible avenue to achieve this may be by considering and acting on adolescents’ views while aiming to increase suitable opportunities and support to facilitate their increased participation. Investigating why these adolescents do not achieve these preferences is outside the scope of this paper, but should be considered in future work. Certain barriers to physical activity such as too much homework as identified previously [45] may prevent these adolescents from achieving these preferences and warrant further investigation.

### Strengths and limitations

This study provides a novel perspective on factors which could inform intervention development and further similar work could be helpful. Although this data cannot provide the depth of insight into these issues as provided by qualitative research, this is a large sample size for data on physical activity preferences and provides a broad overview of adolescent opinion with some tangible suggestions for intervention development. More detailed information regarding the issues explored would only be possible with more specific questions or qualitative work. The activity preference outcome variable asks about which activities or sports adolescents would like to try, or do more often. The aim of this paper was to inform adolescent physical activity promotion, which could practically incorporate both trying new activities and also doing more of activities that are already done. Therefore, we incorporated both elements into the same question aiming not to differentiate between the two as both could lead to increased activity. As we had information on current participation in different types of activity, we were able to see whether adolescents who wanted to do more or try a certain activity were more likely to already participate in that activity. The associated limitation was that we were unable to examine preferences for trying something new and doing more of an activity separately. Nonetheless, this information is useful for informing adolescent physical activity promotion irrespective of the types of activities already participated in by adolescents. The preferences identified in this paper may be specific to this particular age group; as all students were in the same school year it was not relevant to examine age differences. It is also possible that preferences regarding the timing, location and co-participation of activity may also differ for specific activities; we are unable to investigate this with the current data. Results should be interpreted with caution as the SPEEDY sample has slightly lower levels of overweight and obesity than the general population [46] and questions used to derive the outcome variables have not been validated. It is also possible that the context in which the questions were asked (a study about physical activity) and the positive framing of the question might have led participants to over report the activities they would be interested in. It is also possible that social desirability might have influenced the responses. As participants were instructed to take the accelerometers off during water-based activities, physical activity levels of swimmers are likely to have been underestimated. Of the 62 participants assessed by mail, 57 had parent-reported height and weight data. Although results did not differ without these participants included, it is possible that there is some error in this data. As suggested in the literature [47], height error is likely to be small but mean weight error may be greater among girls and those with a greater BMI.

### Implications

Targeting adolescent physical activity promotion by subgroup and providing choice around multiple intervention contexts appears important. These strategies are also appropriate considering increasing autonomy during adolescence. Not all activities and contexts will appeal to all adolescents even in a certain population group. Therefore, researchers could explore innovative ways to incorporate choice of activity type, co-participants, timing and location of physical activity within promotion interventions targeting adolescents. Exposing adolescents to new types and locations of activity could have potential for increasing adolescent physical activity. Using schools to deliver interventions is convenient but being more creative about targeting out of school time when delivering interventions within schools warrants future research. Suggestions for this could include offering incentives for participation outside of school or setting PE homework to be done with parents or friends. Within school, offering adolescents more choice in PE lessons could be facilitated by lending schools equipment for new activities or providing schools with suggestions of new activities using existing equipment. Allowing students to borrow sports equipment throughout the day and allowing after school use of this equipment might also be worth investigating.

## Conclusions

Specific targeting of adolescent physical activity promotion by subgroup, incorporating choice of activity type, co-participants, timing and location of physical activity, may have potential for increasing adolescents’ physical activity. Introducing adolescents to new activities may be especially important as once adolescents have tried an activity type they may be more likely to want to do more of it.

## Competing interest

The authors have no competing interest to disclose.

## Authors’ contributions

KC developed the research question, supervised and coordinated data collection, carried out the initial analyses, drafted the initial manuscript, critically reviewed the manuscript, and approved the final manuscript as submitted. AA contributed to manuscript drafting, reviewed and revised the manuscript, and approved the final manuscript as submitted. EvS and UE were involved with the conceptualization and design of the SPEEDY study, reviewed and revised the manuscript, and approved the final manuscript as submitted.

## Pre-publication history

The pre-publication history for this paper can be accessed here:

http://www.biomedcentral.com/1471-2458/13/718/prepub
